# Efficacy of intradermal injection of tranexamic acid and ascorbic acid versus tranexamic acid and placebo in the treatment of melasma: A split‐face comparative trial

**DOI:** 10.1002/hsr2.537

**Published:** 2022-03-09

**Authors:** Nader Pazyar, Seyedeh Nasrin Molavi, Parisa Hosseinpour, Maryam Hadibarhaghtalab, Seyedeh Yasamin Parvar, Motahareh Babazadeh Dezfuly

**Affiliations:** ^1^ Dermatology Department Ahvaz Jundishapur University of Medical Sciences Ahvaz Iran; ^2^ Dermatology Department, Emam Hospital, School of Medicine Ahvaz Jundishapur University of Medical Sciences Ahvaz Iran; ^3^ School of Medicine, Islamic Azad University Kazeroun Branch Kazeroun Iran; ^4^ Molecular Dermatology Research Center Shiraz University of Medical Sciences Shiraz Iran; ^5^ Student Research Committee Shiraz University of Medical Sciences Shiraz Iran

**Keywords:** ascorbic acid, MASI score, melasma, split‐face injection, tranexamic acid

## Abstract

**Background and Aims:**

Melasma is a common dermatologic disorder characterized by symmetrical hyperpigmented lesions on the face. Although various therapeutic options are available for melasma, its treatment remains challenging. The present study evaluated the safety and efficacy of intradermal microinjection of tranexamic acid (TA) plus ascorbic acid in treating melasma lesions compared with TA and placebo.

**Methods:**

From September 2019 to May 2020, 24 patients with symmetrical melasma were enrolled in a prospective, double‐blind, split‐face, randomized controlled clinical trial. Each patient received 50 mg/ml TA and 50 mg/ml ascorbic acid for one side of the face (A) and 50 mg/ml TA and placebo for the other side (B) every 2 weeks for 12 weeks. The Melasma Area and Severity Index (MASI) score, Physician Global Assessment, and pain were measured at baseline and at 4, 8, 12, and 24 weeks. Statistical analysis was done using SPSS software version 16, and data were reported as mean ± standard deviation or median and interquartile range. *χ* ^2^ and Fisher's exact tests were used to test differences between the groups.

**Results:**

Both groups experienced a significant decrease in MASI scores compared with the baseline. The MASI score was significantly less in the intervention group than the placebo group at the 8th and 12th weeks. However, burning pain was significantly more prominent in the intervention group.

**Conclusion:**

Intradermal injection of ascorbic acid combined with TA can be beneficial in treating melasma. Currently, there are numerous treatment modalities for melasma. However, the results still vary, and satisfactory outcomes are yet to be reached.

## 1 INTRODUCTION

Melasma is a common skin issue that results from melanogenesis dysfunction. It is particularly prominent in middle‐aged women and is characterized by dark, hyperpigmented patches. The exact etiology of the disease is still unknown. However, factors like oral contraceptives, steroids, exposure to sunlight, pregnancy, hormone replacement therapy, ovarian tumors, and a stressful lifestyle may promote the development of the disease.^1^, ^2^ Therefore, the emergence of melasma is influenced by various factors and depends on environmental interactions, hormonal effects, and genetic susceptibility. Although melasma is found in all races, it is more prevalent in darker skin phototypes (Fitzpatrick skin III−V); it mainly affects people of Asian, Middle Eastern, Latin American, African, or Hispanic descent.^3^

Even though melasma is primarily a cosmetic concern, this condition can dramatically impact the quality of life of affected patients and causes depression and frustration, reducing their psychosocial quality of life. In 2003, Balkrishnan and colleagues developed and evaluated the Melasma Quality of Life Index. It was reported that 65% of patients felt discomfort due to spots on their face, while 55% felt frustrated and 57% felt ashamed of their skin condition.^4^ A literature review by Pawaskar et al.^5^ also found that melasma has a severe impact on health‐related quality of life and deleteriously affects patients' emotional wellbeing, social life, and physical health.

Nowadays, various treatment modalities are used to eliminate the lesions and prevent recurrences, including systematic and topical agents along with light‐based therapies and lasers.^6^ Broad‐spectrum sunscreens, retinoic acid (tretinoin), azelaic acid, ascorbic acid, tranexamic acid (TA),^7^ and chemical peels (such as salicylic acid and glycolic acid) are other treatment options have been described in the literature.^8^ TA acts as an antifibrinolytic agent by inhibiting the tissue plasminogen activator. It has been shown to reduce blood loss and transfusion requirements in surgical procedures, including emergency trauma surgery, cardiovascular and spinal cord surgery, and hip and knee arthroplasty procedures. TA is also the only FDA‐approved drug for heavy menstrual bleeding. The amount of TA used to treat melasma is much less than the antifibrinolytic dose. The hypopigmentation effect of TA is due to its antiplasmin activity.^9^, ^10^, ^11^, ^12^

According to the comparative study of the efficacy of intradermal and topical TA versus a triple combination of tretinoin 0.025%, hydroquinone 2%, and fluocinolone acetonide 0.01%, intradermal TA led to a significant decrease in the Melasma Area and Severity Index (MASI) score compared with the other groups.^13^ In a split‐face controlled trial by Saki et al.,^14^ a monthly intradermal injection of TA was associated with significantly reduced melanin content compared with topical hydroquinone during the first 4 weeks of treatment. However, after 20 weeks, the overall changes were not significant.

Topical and intradermal injection of ascorbic acid is another option for treating melasma lesions. Espinal‐Perez et al. conducted a double‐blind, randomized controlled trial evaluating the effect of 5% ascorbic acid versus 4% hydroquinone in treating melasma. In this study, both treatments showed satisfactory results without statistical differences in colorimetric measures. Nevertheless, those who received hydroquinone reported more side effects than those who received ascorbic acid (68.7% vs. 6.2% of patients).^15^

The present study aimed to compare the efficacy and adverse effects of administering 50 mg/ml TA and 50 mg/ml ascorbic acid intradermally versus TA and placebo in treating facial melasma lesions. It should be noted that while TA has direct interactions with aspirin, epinephrine, acetaminophen, and 30 other drugs, it does not directly interact with ascorbic acid according to the Medscape and other approved websites.^16^

## 2 MATERIALS AND METHODS

This prospective, double‐blind, randomized split‐face controlled trial was conducted on patients diagnosed with melasma referring to the dermatology clinic of Ahvaz Imam Khomeini Hospital affiliated with Ahvaz Jundishapur University of Medical Sciences, Ahvaz, Iran. The sample size was measured as 24 patients in each group according to the formula below and a related study by Iraji et al.^17^ (95% confidence interval, 80% power, *α*: 0.02, *β*: 0.04, *S*_1_: 0.8, *S*_2_:1.3, mean 1: 1.5, mean 2: 2.8).

N=(z1−(α2)+z1−β)2 × (σ12+σ22)d2.

We included all females aged 18−50 years who had symmetrical melasma with Fitzpatrick II−IV skin types. To reduce the possibility of selection bias, one side of the face was randomly treated with TA and ascorbic acid, while the other was treated with a placebo. We excluded patients with a history of melasma treatment within the last month; pregnant or lactating women; those who were sensitive to the studied drugs; patients with coagulation disorders; patients using oral contraceptives, anticonvulsants (phenytoin), or anticoagulant medications since the past year; and patients with active herpetic lesions and warts on the face.

This controlled trial was conducted in line with the CONSORT statement from September 2019 to May 2020, and the Ahvaz Jundishapur University of Medical Sciences Ethics Committee approved the study. Participants were given necessary information regarding the study, and written informed consent was obtained before commencing the investigation.

### 2.1 Study implementation

In the beginning, all participants were examined using a wood lamp to determine the melasma type (dermal, epidermal, or mixed). Demographic characteristics including sex, age, predisposing factors (pregnancy, exposure to ultraviolet rays, and oral contraceptives), family history of melasma, skin phenotypes, duration of the disease, type of melasma, affected body parts, and history of previous treatments were recorded.

In the following step, after administering a local anesthetic (lidocaine/prilocaine) and dressing on the face for 60 min, an intradermal injection was performed using a 1 ml syringe with intervals of 1 cm. To keep the distance of injections at 1 cm intervals, we measured the distances with a ruler and marked them with a pen. Thirty minutes after the procedure, the spots were cleaned with an alcohol pad. Each site was injected with 0.1 ml of the solution. Intradermal injection of drugs was performed by injecting the needle at an angle of 5−15° into the skin, leading to the formation of a wheal‐like area on the skin.^18^ TA and ascorbic acid were available in vials of 500 mg per 5 ml solution. In Group A, one side of the face was injected with 0.5 ml of TA (50 mg) and 0.5 ml of normal saline. In Group B, the other side of the face was injected with 0.5 ml of TA (50 mg) and 0.5 ml of ascorbic acid (50 mg). After the injection, patients were advised to apply a cold compress and use sunscreen. Injections were performed in 2‐week intervals for 12 weeks.

### 2.2 Outcome measures

Based on standard guidelines, the MASI was used to evaluate the involved area, darkness, and homogeneity of hyperpigmentation. For calculating the involved area (A), the right side of the forehead, the cheek, and the chin were calculated as 15%, 30%, and 5% of the whole face, respectively. Similar areas on the left side of the face were calculated in the same way, reaching a total of 100%. The final score ranged between 0 and 6 (0 = no involvement, 1 = 0%−9%, 2 = 10%−29%, 3 = 30%−49%, 4 = 50%−69%, 5 = 70%−89% and 6 = 90%−100%). Darkness (D) is evaluated based on a 0−4 scale: scale 0 means the absence of any darkness; scale 1 is a light brown color; scale 2 is a brown color; scale 3 is a dark brown color; scale 4 means black. Homogeneity (H) is measured on a 0−4 scale, from the minimal to the maximal grade of homogeneity. At last, the MASI score is calculated by multiplying the A score by the sum of D and H for each of the six regions. The maximum score for each side is 24, and the minimum is zero.

MASI score: 0.15(A)(D + H) + 0.3(A)(D + H) + 0.05(A)(D + H).

Patients were visited, and the MASI score and potential side effects were evaluated at baseline and after 4, 8, 12, and 24 weeks of treatment.

The Dynamic Physician Global Assessment (Dynamic PGA) was also used to evaluate the response to treatment by taking photographs of the lesions with a digital camera (Samsung Galaxy Note10+) at the beginning and end of the study. In this scale, 0%−25% improvement was reported as poor improvement, 26%−50% and 51%−75% were reported as fair and good improvement, respectively, and 76%−100% improvement in lightening was reported as an excellent improvement. The patients' pain score was also recorded on a scale of 0−10, where 0 indicates no pain and 10 indicates the most severe pain.

### 2.3 Statistical analysis

Statistical analysis was done using SPSS software version 16 (SPSS Inc.). A descriptive analysis was done on the demographic information, and data were reported as mean ± standard deviation (SD) or median and interquartile range (IQR) in case of nonparametric variables (e.g., duration of melasma). Frequencies were presented as numbers and percentages. *χ* ^2^ and Fisher's exact tests were used to test the differences between the categorical variables. A *p *value less than 0.05 was considered significant.

## 3 RESULTS

**Figure 1:**
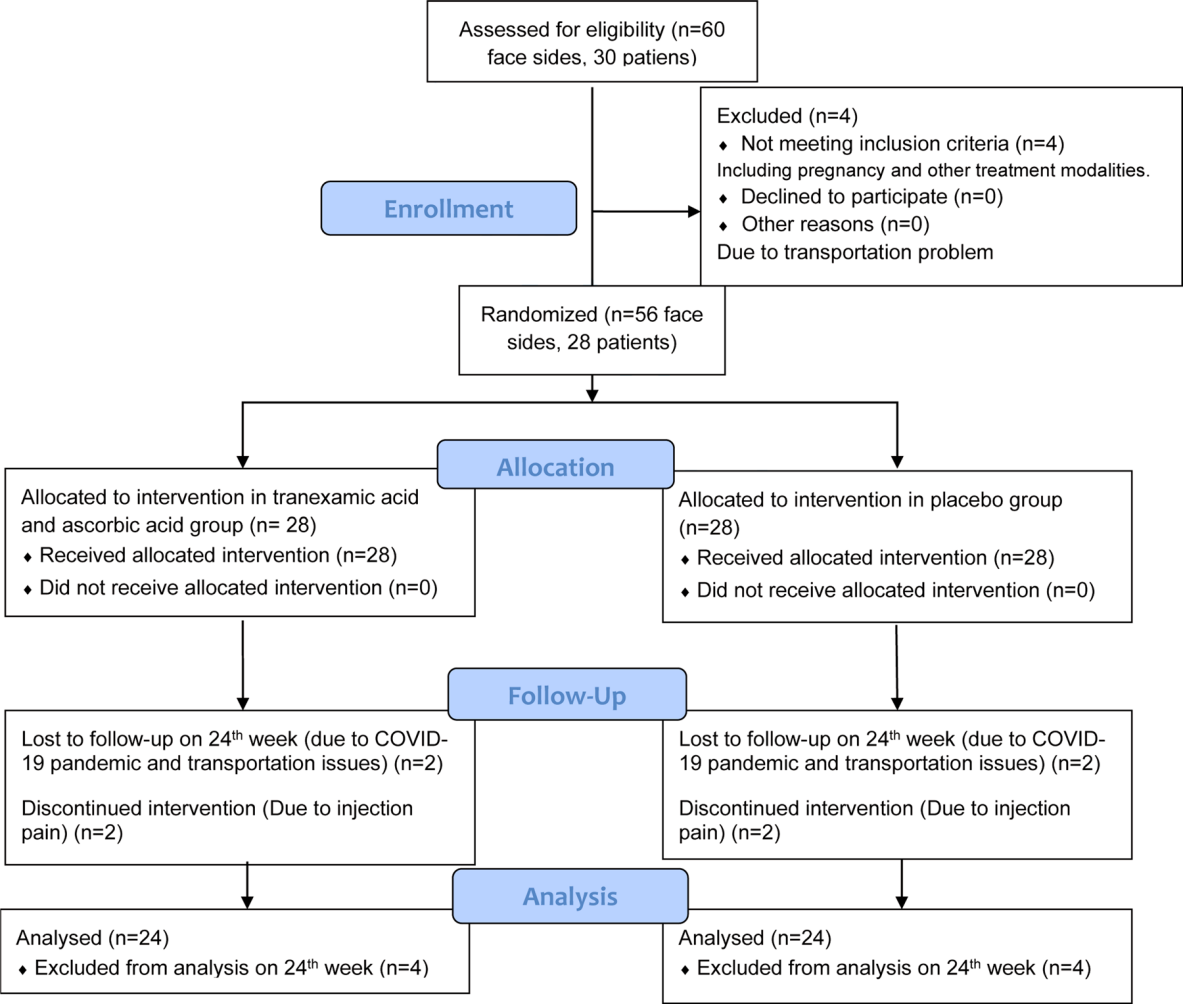
Flow chart of the clinical trial (each number represents one face side of the participants)

Thirty female patients with bilateral symmetrical melasma lesions were eligible for the study (Figure 1). One patient was excluded from the study due to pregnancy, while another was excluded due to using other treatment modalities. Twenty‐eight patients were enrolled in each group. Furthermore, two participants were also lost to follow‐up due to transportation issues and the COVID‐19 pandemic, and two participants discontinued the treatment due to injection pain. Therefore, a total number of 24 patients with 24 sides of the face in each group were injected and analyzed.

The mean age of the studied patients was 35.54 ± 5.26 years, ranging from 28 to 46 years; most patients were in their forties (62.5%). The median duration of the disease in the studied patients was 4.0 (IQR 2.0−6.0) years, ranging from 1 to 12 years. Seventeen patients (70.8%) had a positive family history of melasma, and 16 patients (66.7%) had a positive history of previous treatment. According to the distribution of melasma, eight patients (33.3%) had centrofacial lesions, and the other 16 patients had malar patterns. Eighteen patients (75%) had epidermal melasma 1, 9, and 14 participants had Fitzpatrick skin types II, III, and IV, respectively. Demographic data of patients and melasma features are shown in Table 1.

**Table 1:** Demographic data and melasma features of the study population

Variable	Category	Frequency, *N *(%) or Median [IQR]
Age	20−30	4 (16.7)
	30−40	15 (62.5)
	40−50	5 (20.8)
Duration of melasma (years)	Median (IQR)	4.0 [2.0−6.0]
Family history of melasma, *N *(%)	Positive	17 (70.8)
	Negative	7 (29.2)
Fitzpatrick skin type	Type I	0 (0%)
	Type II	1 (4.2)
	Type III	9 (37.5)
	Type IV	14 (58.3)
Distribution of melasma	Centrofacial	8 (33.3)
	Malar	16 (66.7)
Type of melasma	Epidermal	18 (75.0)
	Mixed	6 (25.0)
Previous treatment	Positive	16 (66.7)
	Negative	8 (33.3)
Predisposing factors	Pregnancy	5 (20.8
	UV	6 (25)
	OCP	4 (16.7)
	OCP + UV	2 (8.3)
	Pregnancy + UV	5 (20.8)
	Pregnancy + UV + OCP	1 (4.2)
	Negative	1 (4.2)

*Note*: Frequencies are reported as number (%) and median [interquartile range (IQR)].

Abbreviation: OCP, oral contraceptive pills.

Evaluation of melasma exacerbating factors showed that one (4.2%) of the patients had no aggravating factors, five (20.8%) had the pregnancy factor. Also, six patients (25%) had the ultraviolet (UV) light exposure factor, four (16.7%) had a history of taking oral contraceptive pills (OCP), five (20.8%) had both pregnancy and UV exposure, and two (8.3%) had both factors of OCP pills and UV exposure. Finally, one person (4.2%) had prior pregnancy, OCP, and UV exposure as exacerbating factors.

**Figure 2:**
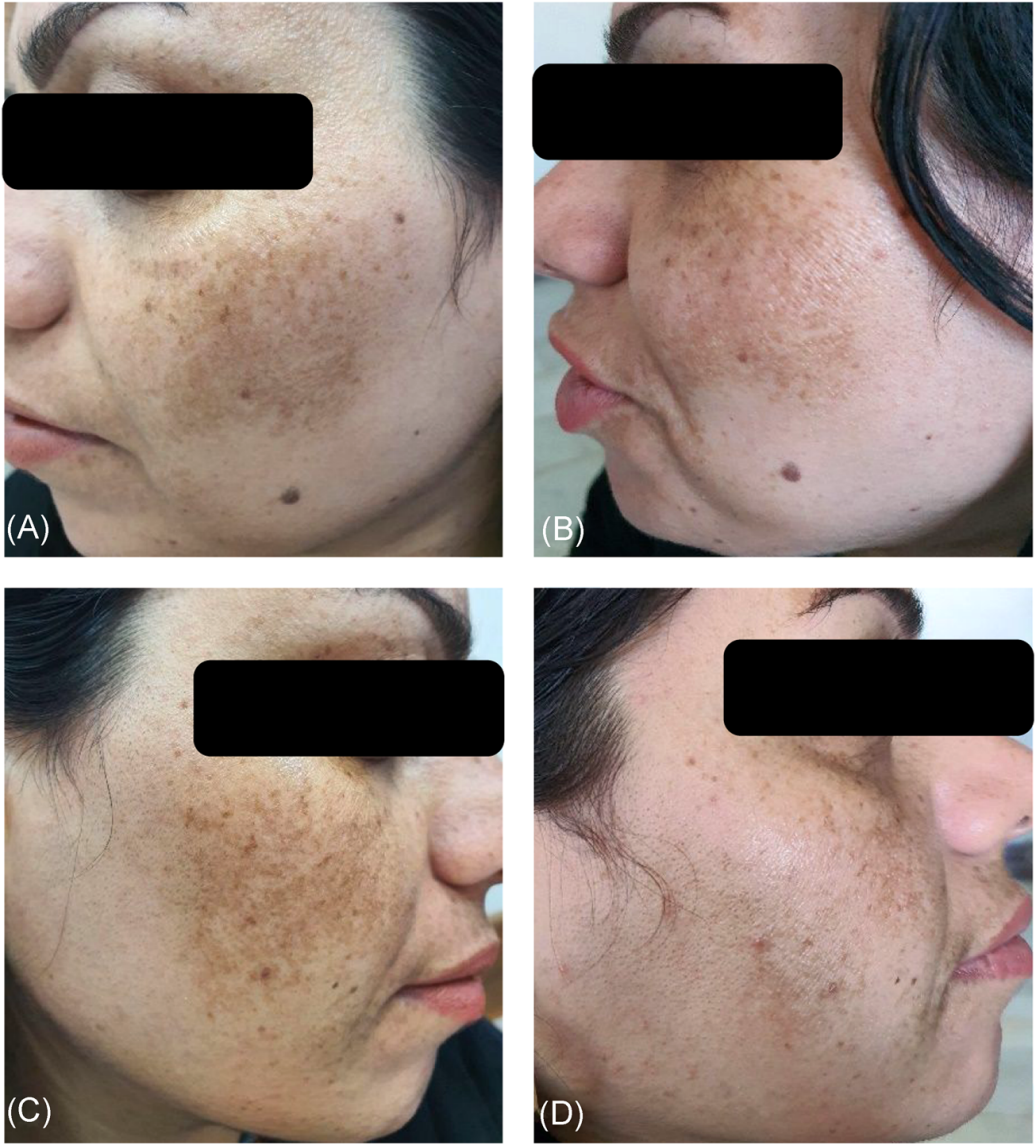
Photographs of the same patient: (A) Before treatment; (B) after treatment with tranexamic acid and placebo; (C) before treatment; (D) after treatment with tranexamic acid and ascorbic acid

**Figure 3:**
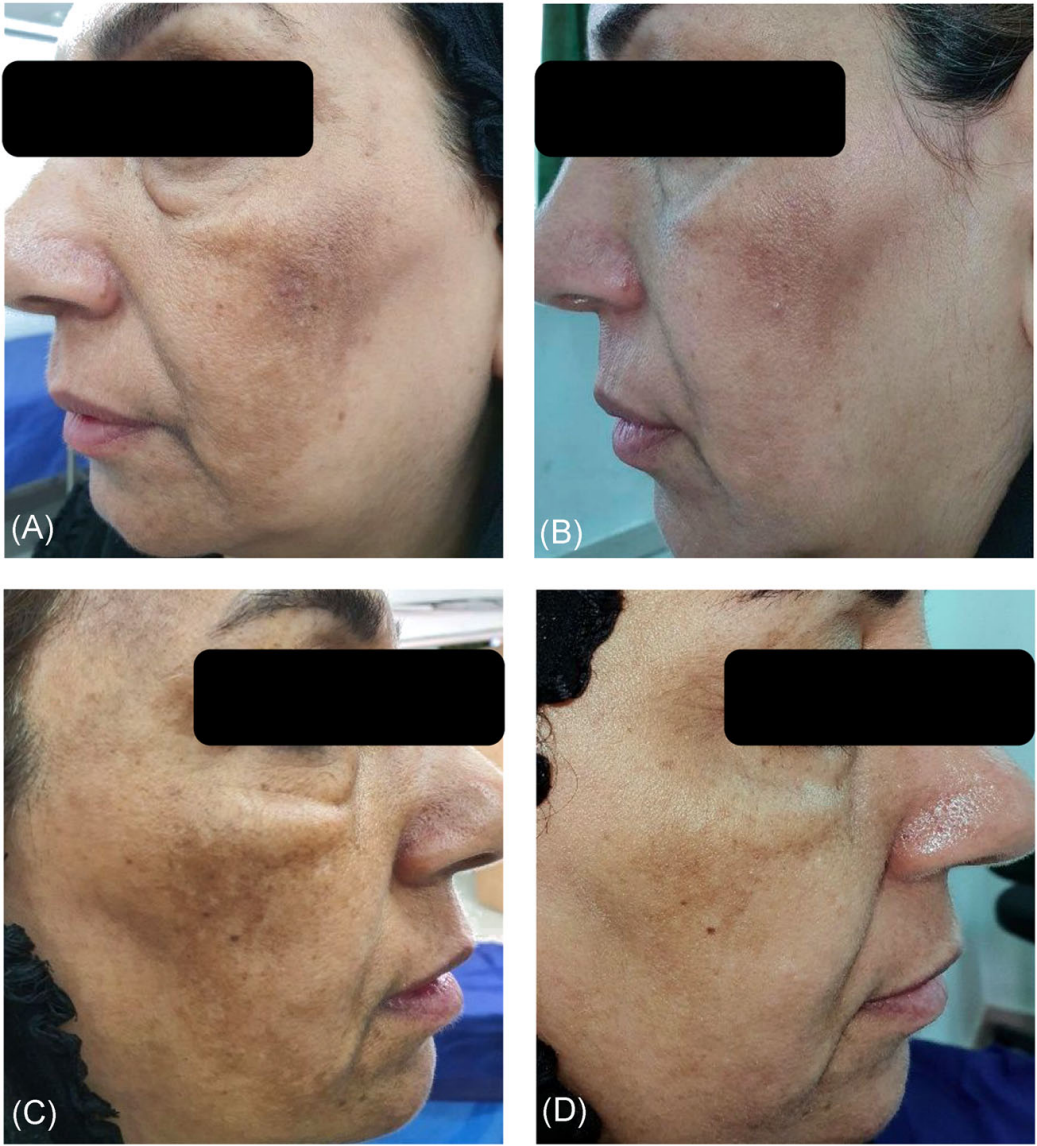
Photographs of the same patient: (A) before treatment; (B) after treatment with tranexamic acid and placebo; (C) before treatment; (D) after treatment with tranexamic acid and ascorbic acid

A comparison of the effectiveness of treatment according to the MASI score is shown in Table 2. During the study intervals, the MASI score significantly decreased (*p *value between groups < 0.001) from the eighth week onwards (8th, 12th, and 24th weeks compared with the baseline). In general, the average MASI score of patients in Group A decreased by 1.29 points, falling from 4.49 (SD 1.48) at the baseline to 3.20 (SD 1.21) 24 weeks later. This is while the average MASI score of patients in the intervention group decreased by 2 points from 4.61 (SD 1.54) at the baseline to 2.61 (SD 1.14) 24 weeks later. It is also worth mentioning that there were statistically significant differences in both groups in each follow‐up compared with the last one (*p* < 0.001). Figures 2 and 3 show both sides of the face of two patients before treatment and 24 weeks later.

**Table 2:** Comparison of the effectiveness of the intervention according to the Melasma Area and Severity Index (MASI) score in groups A (tranexamic acid plus normal saline) and B (tranexamic acid plus ascorbic acid)

Timeline	Group (*N *= 24)	Mean ± SD	*p *value
Baseline	A	4.49 ± 1.48	0.30
	B	4.61 ± 1.54	
4th week	A	4.29 ± 1.44	0.63
	B	4.34 ± 1.51	
8th week	A	3.66 ± 1.35	**<0.001**
	B	2.40 ± 1.25	
12th week	A	2.82 ± 1.29	**<0.001**
	B	2.40 ± 1.25	
24th week	A	3.20 ± 1.21	**<0.001**
	B	2.61 ± 1.14	

*Note*: Bold *p* values are statistically significant at 0.05%.

Abbreviation: SD, standard deviation.

**Figure 4:**
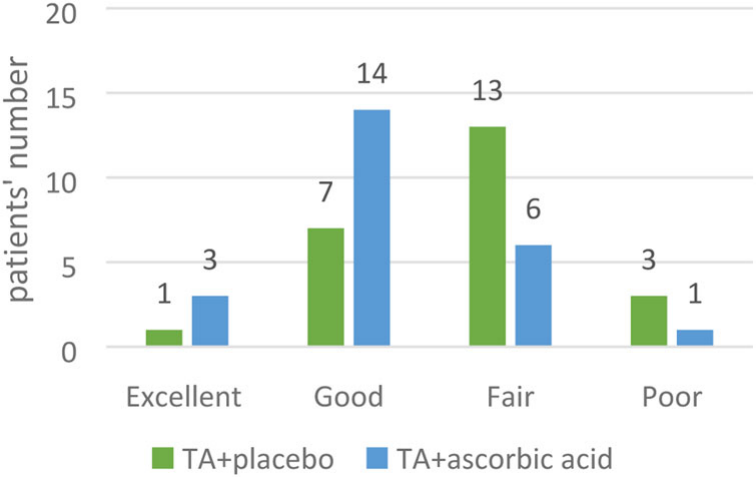
Physicians Global Assessment (PGA) in melasma patients injected with tranexamic acid (TA) plus placebo versus TA plus ascorbic acid

The Physician Global Assessment (PGA) rated the improvement in lesions in the tranexamic acid (TA) plus placebo group as excellent in 1 patient, good in 7, fair in 13, and poor in 3 patients. Improvement in the TA plus ascorbic acid group was rated excellent in 3, good in 14, fair in 6, and poor in 1 patient (Figure 4). The results showed a statistically significant difference between the two groups (*p *= 0.003).

According to the treatment complications in the studied patients, all patients in both groups reported pain and burning sensation in the injection site despite topical anesthesia. The mean pain score of patients in the ascorbic acid group was significantly higher at 0.82 (SD 0.11), while in the placebo group, the mean pain score was 0.50 (SD 0.10) (*p* = 0.01).

## 4 DISCUSSION

Melasma is an acquired condition of the facial skin that mainly affects childbearing women. Clinically, it commonly affects the face in a centrofacial pattern of patches and macules with irregular borders. The course of the disease is resistant to treatment and has a high recurrence rate; therefore, treatment of melasma still requires more evidence and a combination of effective pharmacological and nonpharmacological interventions.^6^

Ascorbic acid is one of the suggested supplements in managing patients with melasma. It acts by chelating copper ions used in the cellular pigmentation process, thereby inhibiting melanogenesis.^19^ This study aimed to evaluate the effectiveness of intradermal injection of a combination of TA with ascorbic acid on one side of the face and compared it with the intradermal injection of TA plus placebo on the opposite side in the treatment of melasma. The findings showed that intracutaneous injection of a combination of TA with ascorbic acid could significantly reduce the patients' MASI scores from the eighth week onward compared with the pre‐intervention condition.

In the present study, we injected ascorbic acid intradermally for its accelerated effect, whereas topical ascorbic acid has limited penetration in the skin and may cause extreme dryness.^20^ In another study, Ismail et al. conducted a clinical trial on 30 female patients in Greece on microneedling with topical ascorbic acid in treating melasma. Microneedling followed by applying topical l‐ascorbic acid 20% in each session resulted in a significant decrease in the MASI score from three sessions onward without any serious side effects, especially in patients with Fitzpatrick skin phototypes I−III. In line with our study, the mentioned study tested ascorbic acid with higher concentrations.^21^

To date, no single therapy has been proven to be effective in treating melasma. In contrast, combination therapies (e.g., tretinoin plus corticosteroids plus hydroquinone) appear to be more beneficial than monotherapy.^22^, ^23^ Ali Balvei et al., in a clinical trial in Australia, evaluated the effect of a combination of mesotherapy with ascorbic acid and salicylic acid (as a peeling agent) in one group and salicylic acid alone in the other group. After 2 months of therapy with 2‐week intervals between sessions, those who received ascorbic acid mesotherapy experienced a greater decrease in MASI scores than those who received only the peeling agent. However, the reduction was not statistically significant. The authors suggested that long‐term intermittent maintenance therapy and additional treatment sessions could be more effective in prolonging the activity of ascorbic acid in the treatment of melasma.^20^ A clinical trial by Surabhi Dayal et al. on patients with epidermal melasma evaluated 20% trichloroacetic acid versus combination therapy of 5% ascorbic acid cream and trichloroacetic acid. The researchers showed that although both study groups experienced a significant reduction in MASI score, combination therapy also led to a significant reduction in MASI score. Furthermore, those who received trichloroacetic acid experienced more side effects, though this was not significant.^7^ Although the study used ascorbic acid topically, a finding similar to the present study was the effectiveness of adding ascorbic acid to the patient's medication regimen and using a combination treatment.

Recent studies conducted in Iran share a similar population to that of ours and facilitate a more accurate comparison. Iraji and his colleagues, in a comparative study conducted on 30 patients with melasma, evaluated the effect of mesotherapy with ascorbic acid and TA with and without glutathione. At the end of the study, both groups experienced a significant decrease in the MASI score relative to the baseline scores.^17^

Like other injection methods, pain and burning were common side effects of our treatment in all patients, though the ascorbic acid group reported significantly more degrees of pain. In contrast, a study by Liliana Elizabeth Espinal Perez et al. comparing the effect of standard hydroquinone treatment with vitamin C found that vitamin C had fewer adverse effects.^15^ A mild to moderate burning sensation, erythema, ecchymosis, and edema are other reported adverse effects of vitamin C in the literature.^17^, ^20^

Our study had several limitations. First of all, if the sample size had been larger, the results would be more accurate. Since enrollment in the study required consent and multiple sessions of in‐person visits were mandatory, access to a larger sample size was challenging. Furthermore, some patients did not show up for the last follow‐up. Secondly, there may be selection bias in assessing patients with facial melasma. Another limitation of this study was that we only used the MASI score for evaluating melasma. This is while other modalities, including Visioface® photography and the Mexameter®, can allow a more comprehensive analysis of melasma lesions.

## 5 CONCLUSION

The intradermal injection of a combination of TA and ascorbic acid could significantly reduce patients' MASI scores, and this improvement was sustained until 3 months. Currently, there are numerous treatment modalities for melasma. However, the results still vary, and satisfactory outcomes are yet to be reached. Thus, more research is needed to find the most efficient treatment.

## CONFLICTS OF INTEREST

The authors declare no conflicts of interest.

## ETHICS STATEMENT

The Ethics Committee of the Ahvaz Jundishapur University of Medical Sciences approved the present study under code number IR.AJUMS.REC.1398.623. The study was also enrolled in and approved by the Iranian Registry of Clinical Trials (IRCT20191213045719N1). Written informed consent was obtained from all patients before initiating the trial. All patients were guaranteed that their information would be kept confidential.

## AUTHOR CONTRIBUTIONS

*Conceptualization, supervision*: Nader Pazyar. *Conceptualization and formal analysis*: Seyedeh Nasrin Molavi. *Methodology and writing—original draft*: Parisa Hosseinpour and Maryam Hadibarhaghtalab. *Supervision, writing—original draft and writing—review and editing*: Seyedey Yasamin Parvar. *Methodology and supervision*: Motahareh Babazadeh Dezfuly.

## Data Availability

The data that support the findings of this study are available from the corresponding author upon reasonable request.
